# Survey data of internet skills, internet attitudes, computer self-efficacy, and digital citizenship among students in Indonesia

**DOI:** 10.1016/j.dib.2021.107569

**Published:** 2021-11-16

**Authors:** Wibowo Heru Prasetiyo, Noor Banu Mahadir Naidu, Beti Indah Sari, Rochman Hadi Mustofa, Naillysa Rahmawati, Gilang Pambudi Adi Wijaya, Obby Taufik Hidayat

**Affiliations:** a Civic Education Department, Universitas Muhammadiyah Surakarta, Surakarta, Indonesia; b Moral, Civics, and Character Building Studies, Universiti Pendidikan Sultan Idris, Tanjong Malim, Malaysia; c Social Science Education, IAIN Tulungagung, Tulungagung, Indonesia; d Accounting Education, Universitas Muhammadiyah Surakarta, Surakarta, Indonesia

**Keywords:** Digital citizenship, Internet skills, Internet attitudes, Computer self-efficacy

## Abstract

Dataset provides wide measurement data on internet skills, internet attitudes, computer self-efficacy of the students related to the digital citizenship in Indonesia. Due to the pandemic of Covid-19, the survey was conducted online by considering the informant consent on assessing demographic information (7 items), internet skills (9 items), internet attitude (5 items), expertise and skills in using a computer (5 items), respect (6 items), educate (5 items), and protect (4 items), which was carried out from March to April. There were a total of 581 respondents selected through probability sampling based on random convenient sample from 12 public and private senior high schools which spread throughout 5 cities in Central Java, Indonesia. The survey data were analyzed using multivariate analysis and partial least structure with the analysis technique of Structural Equation Modelling (SEM). In the future, this data can help educators, researchers, and educational policy makers to determine the level of readiness of students' digital citizenship attributes and efforts to conduct further research on efforts to strengthen digital citizenship in curricular programs.

## Specifications Table

**Table:** 

Subject	Social Sciences
Spesific subject area	Education Citizenship education
Type of data	Primary data, Tables
How data were acquired	Data were acquired through online platform survey (Google Form) at the selected schools. The questionnaire is provided as a supplementary file. The acquired data were imported to Excel and analyzed using SmartPLS 3 software.
Data format	Raw, analyzed, and descriptive data
Parameters for data collection	The survey data were acquired from 581 students in the period of March-April 2021
Description of data collection	The help of probability sampling and random convenient sample combination was used to select the respondents through Google Form at 12 schools in Central Java, Indonesia
Data source location	Survey was conducted in the 5 cities are Surakarta, Karanganyar, Boyolali, Klaten, and Sukoharjo
Data accessibility	Dataset is uploaded on Mendeley Repository name: Mendeley Data identification number: https://dx.doi.org/10.17632/2rvzyk3rjd.2 Direct URL to data: https://data.mendeley.com/datasets/2rvzyk3rjd/1

## Value of the Data

• The dataset helps provide empirical evidence on digital citizenship readiness among students in Indonesia. The main attributes of digital citizenship include respect, educate, and protect (REP) and the factors that have an impact on digital citizenship are also presented.
• The dataset will be useful for the researchers who want to make comparison with similar research, specifically the preparation for the digital citizenship aspect to face the era of Society 5.0.
• The dataset can be used to enlighten Indonesian educators and educational policymakers in taking further efforts to equip students with the knowledge of how to respond to the abundance and ease of access to technology in a responsible manner, especially in the aspects of technology literacy and ethics when using the internet.

## 1 Data Description

The data in this article provides in-depth information about the level of digital citizenship readiness of Indonesian students by looking at the factors such as internet skills, internet attitudes, and computer-self efficacy. The survey in this study involved 581 students that spread across 12 senior high school consisted of private and public school in Central Java, Indonesia. The focus of data collection consists of three groups of variables, including (A) Demography and Socio-Economy of individuals, including gender, age, school of origin, parents' educational background, frequency of internet usage in one day, types of gadgets used in accessing the internet, and the monthly internet budget that the students spend in using the internet. (B) The variable of computer and internet competence which includes the ability and expertise of students in using/operating a computer which are outlined in 19 question items that are aimed at measuring students' internet skills, internet attitudes, and self-efficacy. And (C) 15 question items to measure the level of digital citizenship of students based on three main components, namely respect, educate, and protect (REP). All question items used to measure the two variables of the computer group, internet competence, and student digital citizenship were measured using a Likert scale with 5-point intervals (Strongly Agree to Strongly Disagree) which are presented in Table 1, Table 2, Table 3, Table 4, Table 5, Table 6, Table 7, Table 8.

**Table 1:** Demographic and socioeconomic characteristics (*n* = 581).

Variable	Category	Frequency	Percentage (%)	Mean	Median	Std. Deviation	Variance	Range	Min	Max	Sum
School Type	Public	374	64.4	1.36	1.00	0.479	0.230	1	1	2	788
	Private	207	35.6								
Gender	Male	144	24.8	1.75	2.00	0.432	0.187	1	1	2	1018
	Female	437	75.2								
Age of Respondents	16	285	49.1	1.59	2.00	0.655	0.429	5	1	6	922
	17	257	44.2								
	18	35	6.0								
	19	3	0.5								
	20	0	0								
	>20	1	0.2								
Parents’ Educational Background	Elementary School	74	12.7	2.90	3.00	1.057	1.118	5	1	6	1687
	Junior High School	100	17.2								
	Senior High School	245	42.2								
	Bachelor's Degree	135	23.2								
	Master's Degree	24	4.1								
	Doctoral Degree	3	0.5								
Frequency of Internet Usage	1-3 hours a day	17	2.9	3.03	3.00	0.848	0.720	3	1	4	1759
	4-6 hours a day	150	25.8								
	7-9 hours a day	214	36.8								
	>9 hours a day	200	34.4								
Gadget Used	Smartphone	337	58.0	2.66	1.00	1.964	3.859	4	1	5	1546
	Tablet	1	0.2								
	Laptop	4	0.7								
	Desktop/ PC	0	0								
	More than one gadget	239	41.1								
Monthly Internet Budget	Rp 10,000 - Rp 25,000	48	8.3	2.89	3.00	0.932	0.868	3	1	4	1678
	Rp 26,000 - Rp 50,000	144	24.8								
	Rp 51,000 - Rp 75,000	214	36.8								
	>Rp 75,000	175	30.1

**Table 2:** Statistic descriptive on internet skill, internet attitude, computer self-efficacy, dan digital citizenship (*n* = 581).

		Mean		95% Confidence Interval for Mean							Skewness		Kurtosis	
Variable	Item	Statistic	Std. Error	Lower Bound	Upper Bound	Var	Std. Dev.	Min	Max	Range	Stat	Std. Error	Stat	Std. Error
Internet Skill	I can download photos/videos on the internet	4.29	0.036	4.22	4.36	0.746	0.864	1	5	4	-1.299	1.853	0.101	0.202
	I know how to use shortcut keys when operating a computer/laptop (e.g., CTRL+C for Copy, CTRL+S for Save)	3.85	0.040	3.77	3.93	0.944	0.971	1	5	4	-0.549	-0.215	0.101	0.202
	I can set the password on laptop/smartphone by myself (Fingerprint, Pattern, Face Unlock)	4.44	0.032	4.38	4.50	0.581	0.762	1	5	4	-1.426	2.279	0.101	0.202
	I can set the Wi-Fi connection with a laptop/smartphone by myself	4.26	0.036	4.19	4.33	0.772	0.879	1	5	4	-1.220	1.393	0.101	0.202
	I find it difficult to search for the right keywords when browsing/googling	2.49	0.045	2.40	2.58	1.188	1.090	1	5	4	0.305	-0.542	0.101	0.202
	I find it difficult to search for the website that I have accessed	2.44	0.044	2.35	2.52	1.146	1.071	1	5	4	0.249	-0.690	0.101	0.202
	I can edit images/photos/videos/music downloaded on the internet	3.73	0.041	3.65	3.81	0.995	0.997	1	5	4	-0.543	-0.105	0.101	0.202
	I can install applications on a smartphone/laptop by myself	4.41	0.031	4.35	4.48	0.550	0.742	1	5	4	-1.321	2.243	0.101	0.202
	I can check how much internet data is used on smartphone applications	4.27	0.038	4.19	4.34	0.837	0.915	1	5	4	-1.216	1.018	0.101	0.202
Internet Attitude	Internet can reduce many difficulties in finishing assignments	4.20	0.038	4.13	4.28	0.841	0.917	1	5	4	-1.247	1.537	0.101	0.202
	Internet brings us to a more advanced era	4.30	0.031	4.24	4.36	0.540	0.735	1	5	4	-0.742	0.065	0.101	0.202
	Life becomes easier and faster with the presence of internet	4.28	0.031	4.22	4.34	0.555	0.745	1	5	4	-0.814	0.500	0.101	0.202
	Internet provides an easy, abundant, and enjoyable source of information	4.34	0.030	4.29	4.40	0.519	0.721	1	5	4	-0.900	0.602	0.101	0.202
	There are many changes in human life which have never been thought previously due to internet applications	4.32	0.031	4.26	4.38	0.552	0.743	1	5	4	-0.969	1.118	0.101	0.202
Computer Self-Efficacy	I am confident in my capability to use computer/laptop to write letters, papers, and other assignments	3.91	0.037	3.84	3.98	0.807	0.898	1	5	4	-0.650	0.423	0.101	0.202
	I am confident in my capability to install and uninstall various computer software	3.45	0.041	3.37	3.53	0.954	0.977	1	5	4	-0.176	-0.263	0.101	0.202
	I am fully confident in my capability to understand some terms related to computer hardware and software (e.g., CPU, hard disk, Operating System, MS. Office)	3.27	0.040	3.19	3.34	0.933	0.966	1	5	4	-0.194	-0.154	0.101	0.202
	I am confident in my capability to improve the skill in operating some computer software (video editing, Photoshop, Corel Draw, KineMaster, VivaVideo, Filmora)	3.39	0.041	3.31	3.47	0.959	0.979	1	5	4	-0.173	-0.258	0.101	0.202
	I am confident in my capability to solve troubleshooting (no response/hang/shut down) when using computer/laptop	2.85	0.042	2.77	2.93	1.023	1.011	1	5	4	0.009	-0.154	0.101	0.202
Respect	I believe that creating any computer virus and sending any spam email are parts of cybercrime	3.92	0.045	3.83	4.00	1.198	1.095	1	5	4	-0.869	0.163	0.101	0.202
	I understand that using technology can cause health and mental disorders such as internet addiction and stress	4.07	0.038	4.00	4.15	0.841	0.917	1	5	4	-0.928	0.761	0.101	0.202
	I believe that hacking other people's emails/social media accounts, downloading music/film illegally, and plagiarizing other people's work without permission are unethical behavior	4.41	0.036	4.34	4.48	0.743	0.862	1	5	4	-1.565	2.241	0.101	0.202
	I believe that everyone has to be responsible in everything they do on the internet (online)	4.49	0.030	4.43	4.54	0.509	0.713	1	5	4	-1.364	1.897	0.101	0.202
	I believe that all internet users have the same right to give opinion and to express themselves	4.29	0.032	4.23	4.36	0.604	0.777	1	5	4	-1.113	1.710	0.101	0.202
	Smartphone and computer make it easier for me to communicate with friends	4.52	0.029	4.47	4.58	0.481	0.694	1	5	4	-1.524	2.676	0.101	0.202
Educate	Before deciding to buy something online, I always try to find out the information about the seller and the goods offered	4.40	0.030	4.35	4.46	0.514	0.717	1	5	4	-1.114	1.344	0.101	0.202
	I love shopping through e-commerce (Shopee, Lazada, Tokopedia, Bukalapak, etc)	3.63	0.044	3.55	3.72	1.122	1.059	1	5	4	-0.388	-0.284	0.101	0.202
	I use social media to express my thoughts, feelings, and things that I experience	3.33	0.040	3.25	3.40	0.913	0.955	1	5	4	-0.047	-0.024	0.101	0.202
	I like learning new things through internet/social media (Youtube, Instagram, Facebook, Twitter, etc)	4.23	0.032	4.16	4.29	0.582	0.763	1	5	4	-0.686	0.017	0.101	0.202
	I spend time on social media such as Facebook and Instagram to get information updates	3.57	0.040	3.49	3.65	0.908	0.953	1	5	4	-0.387	0.134	0.101	0.202
Protect	I regularly change the password on the smartphone/laptop to protect privacy	2.94	0.044	2.85	3.03	1.148	1.072	1	5	4	0.041	-0.477	0.101	0.202
	I always visit reliable and safe websites	3.73	0.038	3.65	3.80	0.829	0.911	1	5	4	-0.508	0.434	0.101	0.202
	I install anti-virus and internet protection on the computer/laptop	3.71	0.040	3.63	3.79	0.934	0.967	1	5	4	-0.460	-0.100	0.101	0.202
	I always read privacy statement before installing new applications	3.76	0.038	3.68	3.83	0.844	0.918	1	5	4	-0.435	0.010	0.101	0.202

**Table 3:** Statistic descriptive on demographic characteristics of respondents and internet skill (*n* = 581).

		Internet Skill								
Variable	Category	IS1	IS2	IS3	IS4	IS5	IS6	IS7	IS8	IS9
School Type	Public	4.37	3.98	4.43	4.31	3.50	3.60	3.76	4.45	4.33
	Private	4.15	3.63	4.45	4.17	3.53	3.50	3.66	4.35	4.16
	Average	4.29	3.85	4.44	4.26	3.51	3.56	3.73	4.41	4.27
	F Test	9.13	17.58	0.10	3.45	0.13	1.08	1.42	2.63	4.45
	Significant	0.00	0.00	0.75	0.06	0.72	0.30	0.23	0.11	0.04
Gender	Male	4.41	4.06	4.56	4.28	3.58	3.78	3.74	4.44	4.40
	Female	4.26	3.78	4.40	4.25	3.49	3.49	3.72	4.41	4.22
	Rata-rata	4.29	3.85	4.44	4.26	3.51	3.56	3.73	4.41	4.27
	F Test	3.43	8.52	4.38	0.08	0.72	7.68	0.04	0.18	3.83
	Significant	0.06	0.00	0.04	0.78	0.40	0.01	0.84	0.67	0.05
Age of Respondents	16	4.21	3.71	4.41	4.21	3.47	3.52	3.72	4.40	4.25
	17	4.37	3.98	4.45	4.29	3.50	3.56	3.70	4.43	4.30
	18	4.46	4.00	4.60	4.40	3.83	3.83	3.91	4.40	4.14
	19	3.67	4.33	4.67	4.67	3.67	4.00	4.67	4.67	4.67
	20									
	>20	5.00	5.00	5.00	5.00	5.00	5.00	3.00	4.00	5.00
	Average	4.29	3.85	4.44	4.26	3.51	3.56	3.73	4.41	4.27
	F Test	1.94	3.62	0.73	0.90	1.32	1.22	1.15	0.20	0.58
	Significant	0.10	0.01	0.57	0.46	0.26	0.30	0.33	0.94	0.67
Parents’ Educational Background	Elementary School	4.38	3.70	4.26	4.18	3.38	3.36	3.59	4.23	4.09
	Junior High School	4.04	3.57	4.32	4.12	3.16	3.31	3.62	4.34	4.16
	Senior High School	4.27	3.88	4.47	4.26	3.48	3.56	3.76	4.42	4.31
	Bachelor's Degree	4.42	4.02	4.49	4.39	3.76	3.76	3.81	4.52	4.30
	Master's Degree	4.58	4.17	4.92	4.33	4.13	4.00	3.75	4.58	4.50
	Doctoral Degree	4.67	4.67	4.67	4.67	4.67	4.67	4.67	4.67	4.67
	Average	4.29	3.85	4.44	4.26	3.51	3.56	3.73	4.41	4.27
	F Test	3.22	3.92	3.54	1.43	6.25	4.11	1.24	2.00	1.40
	Significant	0.01	0.00	0.00	0.21	0.00	0.00	0.29	0.08	0.22
Frequency of Internet Usage	1-3 hours a day	4.12	3.76	4.35	3.88	3.12	3.12	3.71	4.35	3.94
	4-6 hours a day	4.20	3.69	4.34	4.19	3.51	3.54	3.63	4.29	4.26
	7-9 hours a day	4.24	3.92	4.41	4.23	3.43	3.52	3.74	4.40	4.21
	>9 hours a day	4.44	3.91	4.56	4.37	3.64	3.67	3.79	4.53	4.36
	Average	4.29	3.85	4.44	4.26	3.51	3.56	3.73	4.41	4.27
	F Test	3.06	1.94	2.57	2.46	2.05	1.71	0.72	2.90	1.69
	Significant	0.03	0.12	0.05	0.06	0.11	0.16	0.54	0.03	0.17
Gadget Used	Smartphone	4.16	3.64	4.38	4.15	3.45	3.41	3.63	4.36	4.18
	Tablet	5.00	4.00	5.00	5.00	5.00	5.00	5.00	5.00	5.00
	Laptop	4.75	4.25	4.50	4.25	2.50	3.75	4.50	4.75	4.25
	Desktop/ PC									
	More than one gadget	4.48	4.15	4.52	4.41	3.60	3.77	3.85	4.49	4.39
	Average	4.29	3.85	4.44	4.26	3.51	3.56	3.73	4.41	4.27
	F Test	7.21	14.28	1.68	4.59	2.61	6.21	3.68	1.84	2.72
	Significant	0.00	0.00	0.17	0.00	0.05	0.00	0.01	0.14	0.04
Monthly Internet Budget	Rp 10,000 - Rp 25,000	4.33	3.77	4.50	4.33	3.52	3.60	3.94	4.44	4.25
	Rp 26,000 - Rp 50,000	4.22	3.86	4.40	4.22	3.48	3.60	3.56	4.35	4.17
	Rp 51,000 - Rp 75,000	4.28	3.79	4.40	4.25	3.50	3.52	3.74	4.41	4.27
	>Rp 75.000	4.36	3.94	4.51	4.29	3.54	3.58	3.79	4.47	4.35
	Average	4.29	3.85	4.44	4.26	3.51	3.56	3.73	4.41	4.27
	F Test	0.72	0.81	1.04	0.32	0.08	0.21	2.31	0.72	0.97
	Significant	0.54	0.49	0.37	0.81	0.97	0.89	0.08	0.54	0.41

**Table 4:** Statistic descriptive on demographic characteristics of respondents and internet attitude (*n* = 581).

		Internet Attitude				
Variable	Category	IA1	IA2	IA3	IA4	IA5
School Type	Public	4.24	4.33	4.34	4.41	4.36
	Private	4.13	4.24	4.19	4.23	4.25
	$\bar{X}$	4.20	4.30	4.28	4.34	4.32
	F Test	2.02	1.88	5.33	7.91	3.03
	Significant	0.16	0.17	0.02	0.01	0.08
Gender	Male	4.25	4.28	4.31	4.29	4.32
	Female	4.19	4.30	4.27	4.36	4.32
	$\bar{X}$	4.20	4.30	4.28	4.34	4.32
	F Test	0.50	0.14	0.28	1.02	0.00
	Significant	0.48	0.71	0.60	0.31	0.98
Age of Respondents	16	4.20	4.30	4.28	4.32	4.26
	17	4.21	4.30	4.31	4.39	4.39
	18	4.17	4.31	4.20	4.20	4.29
	19	3.67	3.33	3.33	4.33	3.67
	20	-	-	-	-	-
	>20	5.00	4.00	5.00	5.00	4.00
	$\bar{X}$	4.20	4.30	4.28	4.34	4.32
	F Test	0.46	1.35	1.63	0.96	1.69
	Significant	0.77	0.25	0.16	0.43	0.15
Parents’ Educational Background	Elementary School	4.23	4.20	4.19	4.22	4.22
	Junior High School	4.13	4.27	4.28	4.32	4.23
	Senior High School	4.13	4.24	4.20	4.38	4.33
	Bachelor's Degree	4.33	4.44	4.44	4.36	4.40
	Master's Degree	4.38	4.46	4.63	4.42	4.38
	Doctoral Degree	4.33	4.67	4.33	4.33	5.00
	$\bar{X}$	4.20	4.30	4.28	4.34	4.32
	F Test	1.14	1.93	3.13	0.65	1.43
	Significant	0.34	0.09	0.01	0.66	0.21
Frequency of Internet Usage	1-3 hours a day	4.06	3.94	3.82	4.06	4.35
	4-6 hours a day	4.15	4.19	4.17	4.25	4.23
	7-9 hours a day	4.17	4.29	4.25	4.29	4.27
	>9 hours a day	4.29	4.42	4.44	4.51	4.44
	Average	4.20	4.30	4.28	4.34	4.32
	F Test	0.99	4.11	6.50	5.74	2.68
	Significant	0.40	0.01	0.00	0.00	0.05
Gadget Used	Smartphone	4.15	4.26	4.25	4.32	4.29
	Tablet	5.00	5.00	5.00	5.00	5.00
	Laptop	4.25	4.00	4.25	4.75	4.25
	Desktop/ PC	-	-	-	-	-
	More than one gadget	4.27	4.36	4.33	4.37	4.36
	$\bar{X}$	4.20	4.30	4.28	4.34	4.32
	F Test	0.97	1.35	0.77	0.97	0.73
	Significant	0.41	0.26	0.51	0.40	0.54
Monthly Internet Budget	Rp 10,000 - Rp 25,000	4.19	4.35	4.35	4.40	4.38
	Rp 26,000 - Rp 50,000	4.28	4.26	4.30	4.35	4.26
	Rp 51,000 - Rp 75,000	4.14	4.26	4.23	4.29	4.32
	>Rp 75.000	4.22	4.35	4.31	4.39	4.35
	$\bar{X}$	4.20	4.30	4.28	4.34	4.32
	F Test	0.73	0.71	0.58	0.77	0.56
	Significant	0.53	0.55	0.63	0.51	0.64

**Table 5:** Statistic descriptive on demographic characteristics of respondents and computer self-efficacy (*n* = 581).

		Computer Self-Efficacy				
Variable	Category	CSE1	CSE2	CSE3	CSE4	CSE5
School Type	Public	3.99	3.50	3.35	3.45	2.89
	Private	3.75	3.35	3.11	3.28	2.78
	Average	3.91	3.45	3.27	3.39	2.85
	F Test	9.73	3.25	8.27	4.12	1.73
	Significant	0.00	0.07	0.00	0.04	0.19
Gender	Male	3.95	3.74	3.56	3.64	3.22
	Female	3.89	3.35	3.17	3.31	2.73
	Average	3.91	3.45	3.27	3.39	2.85
	F Test	0.43	18.25	17.82	12.54	25.76
	Significant	0.51	0.00	0.00	0.00	0.00
Age of Respondents	16	3.83	3.36	3.16	3.37	2.79
	17	4.00	3.51	3.33	3.40	2.88
	18	3.89	3.57	3.49	3.40	3.14
	19	4.00	4.00	4.00	4.33	3.33
	20	-	-	-	-	-
	>20	5.00	5.00	5.00	4.00	3.00
	Average	3.91	3.45	3.27	3.39	2.85
	F Test	1.57	1.76	2.89	0.84	1.21
	Significant	0.18	0.14	0.02	0.50	0.31
Parents’ Educational Background	Elementary School	3.73	3.38	3.11	3.35	2.76
	Junior High School	3.66	3.20	3.00	3.13	2.54
	Senior High School	3.98	3.50	3.33	3.42	2.84
	Bachelor's Degree	4.01	3.52	3.41	3.51	3.10
	Master's Degree	4.08	3.54	3.25	3.46	3.04
	Doctoral Degree	4.67	4.67	4.33	4.33	4.00
	Average	3.91	3.45	3.27	3.39	2.85
	F Test	3.48	2.67	3.52	2.52	4.70
	Significant	0.00	0.02	0.00	0.03	0.00
Frequency of Internet Usage	1-3 hours a day	3.59	3.53	3.41	3.35	3.06
	4-6 hours a day	3.89	3.41	3.27	3.34	2.78
	7-9 hours a day	3.87	3.45	3.27	3.41	2.87
	>9 hours a day	3.99	3.46	3.25	3.42	2.87
	Average	3.91	3.45	3.27	3.39	2.85
	F Test	1.41	0.11	0.15	0.20	0.53
	Significant	0.24	0.96	0.93	0.90	0.66
Gadget Used	Smartphone	3.71	3.29	3.12	3.27	2.72
	Tablet	5.00	5.00	4.00	4.00	5.00
	Laptop	4.25	4.25	4.25	4.00	3.75
	Desktop/ PC	-	-	-	-	-
	More than one gadget	4.18	3.65	3.44	3.55	3.01
	Average	3.91	3.45	3.27	3.39	2.85
	F Test	14.42	8.43	6.88	4.69	6.43
	Significant	0.00	0.00	0.00	0.00	0.00
Monthly Internet Budget	Rp 10.000 - Rp 25.000	3.92	3.42	3.44	3.50	3.06
	Rp 26.000 - Rp 50.000	3.88	3.31	3.21	3.31	2.76
	Rp 51.000 - Rp 75.000	3.95	3.47	3.32	3.36	2.82
	>Rp 75.000	3.89	3.53	3.20	3.46	2.91
	Rata-rata	3.91	3.45	3.27	3.39	2.85
	F Test	0.25	1.41	1.15	0.89	1.32
	Significant	0.86	0.24	0.33	0.44	0.27

**Table 6:** Statistic descriptive on demographic characteristics of respondents and digital citizenship (*n* = 581).

		Digital Citizenship														
		Respect						Educate					Protect			
Variable	Category	R1	R2	R3	R4	R5	R6	E1	E2	E3	E4	E5	P1	P2	P3	P4
School Type	Public	4.00	4.13	4.51	4.56	4.30	4.56	4.45	3.70	3.34	4.26	3.63	2.89	3.76	3.79	3.76
	Private	3.76	3.98	4.23	4.35	4.28	4.44	4.32	3.51	3.30	4.15	3.45	3.03	3.66	3.56	3.74
	$\bar{X}$	3.92	4.07	4.41	4.49	4.29	4.52	4.40	3.63	3.33	4.23	3.57	2.94	3.73	3.71	3.76
	F Test	6.29	3.34	14.27	11.33	0.16	3.99	4.62	4.59	0.23	2.79	5.03	2.15	1.70	7.70	0.10
	Significant	0.01	0.07	0.00	0.00	0.69	0.05	0.03	0.03	0.63	0.10	0.03	0.14	0.19	0.01	0.75
Gender	Male	4.09	3.90	4.32	4.38	4.28	4.46	4.30	3.40	3.33	4.32	3.68	2.88	3.85	3.86	3.67
	Female	3.86	4.13	4.44	4.52	4.30	4.54	4.44	3.71	3.32	4.19	3.53	2.96	3.69	3.66	3.78
	$\bar{X}$	3.92	4.07	4.41	4.49	4.29	4.52	4.40	3.63	3.33	4.23	3.57	2.94	3.73	3.71	3.76
	F Test	4.91	7.31	2.18	4.05	0.02	1.59	4.20	9.20	0.00	2.92	2.68	0.59	3.29	4.76	1.53
	Significant	0.03	0.01	0.14	0.04	0.89	0.21	0.04	0.00	0.99	0.09	0.10	0.44	0.07	0.03	0.22
Age of Respondents	16	3.85	4.07	4.33	4.47	4.27	4.54	4.44	3.64	3.30	4.23	3.56	2.94	3.68	3.62	3.76
	17	4.00	4.08	4.53	4.53	4.33	4.51	4.40	3.64	3.34	4.23	3.56	2.89	3.74	3.81	3.71
	18	3.74	4.03	4.11	4.34	4.26	4.43	4.14	3.60	3.43	4.14	3.66	3.11	4.00	3.60	3.91
	19	4.67	4.00	4.67	4.33	3.67	4.67	4.67	3.67	3.33	4.67	3.67	4.33	3.67	4.33	4.67
	20	-	-	-	-	-	-	-	-	-	-	-	-	-	-	-
	>20	5.00	5.00	5.00	5.00	5.00	5.00	5.00	3.00	3.00	3.00	3.00	5.00	4.00	5.00	5.00
	$\bar{X}$	3.92	4.07	4.41	4.49	4.29	4.52	4.40	3.63	3.33	4.23	3.57	2.94	3.73	3.71	3.76
	F Test	1.40	0.28	3.13	0.76	0.95	0.35	1.61	0.10	0.19	1.01	0.18	2.59	1.02	2.07	1.64
	Significant	0.23	0.89	0.01	0.55	0.44	0.84	0.17	0.98	0.95	0.40	0.95	0.04	0.40	0.08	0.16
Parents’ Educational Background	Elementary School	4.05	4.22	4.51	4.39	4.23	4.50	4.34	3.58	3.32	4.12	3.35	3.19	3.68	3.62	3.86
	Junior High School	3.64	4.12	4.29	4.54	4.26	4.50	4.35	3.48	3.41	4.14	3.53	3.05	3.66	3.47	3.86
	Senior High School	3.90	4.07	4.42	4.49	4.33	4.47	4.43	3.76	3.36	4.23	3.64	2.98	3.73	3.78	3.78
	Bachelor's Degree	4.06	3.92	4.41	4.48	4.27	4.59	4.37	3.56	3.28	4.30	3.55	2.76	3.79	3.77	3.60
	Master's Degree	4.00	4.29	4.54	4.46	4.29	4.75	4.67	3.63	3.04	4.33	3.67	2.50	3.88	3.88	3.46
	Doctoral Degree	4.00	4.33	4.33	4.67	4.67	4.67	5.00	3.67	2.33	4.67	4.67	2.33	3.33	4.33	5.00
	$\bar{X}$	3.92	4.07	4.41	4.49	4.29	4.52	4.40	3.63	3.33	4.23	3.57	2.94	3.73	3.71	3.76
	F Test	2.03	1.51	0.72	0.42	0.40	1.10	1.44	1.25	1.34	1.11	1.93	2.91	0.50	2.10	2.93
	Significant	0.07	0.19	0.61	0.83	0.85	0.36	0.21	0.28	0.25	0.35	0.09	0.01	0.77	0.06	0.01
Frequency of Internet Usage	1-3 hours a day	3.35	3.88	4.12	4.35	3.88	4.24	4.24	3.29	3.06	3.94	3.24	3.29	3.88	3.76	3.76
	4-6 hours a day	3.90	4.06	4.35	4.51	4.29	4.45	4.37	3.51	3.26	4.11	3.38	2.83	3.67	3.67	3.78
	7-9 hours a day	3.94	4.13	4.37	4.45	4.27	4.49	4.39	3.65	3.29	4.16	3.55	3.03	3.76	3.76	3.82
	>9 hours a day	3.95	4.05	4.53	4.52	4.36	4.63	4.46	3.74	3.44	4.41	3.76	2.91	3.73	3.68	3.67
	$\bar{X}$	3.92	4.07	4.41	4.49	4.29	4.52	4.40	3.63	3.33	4.23	3.57	2.94	3.73	3.71	3.76
	F Test	1.61	0.56	2.25	0.58	2.18	3.26	0.76	1.86	1.66	6.27	5.51	1.74	0.41	0.39	0.94
	Significant	0.19	0.64	0.08	0.63	0.09	0.02	0.52	0.13	0.18	0.00	0.00	0.16	0.74	0.76	0.42
Gadget Used	Smartphone	3.81	4.01	4.34	4.43	4.26	4.45	4.35	3.56	3.37	4.15	3.52	3.02	3.67	3.53	3.79
	Tablet	5.00	5.00	5.00	5.00	5.00	5.00	5.00	5.00	5.00	5.00	5.00	2.00	5.00	5.00	5.00
	Laptop	4.50	4.00	4.00	4.50	4.50	4.75	4.75	4.00	4.50	4.50	3.50	2.50	3.00	3.75	3.50
	Desktop/ PC	-	-	-	-	-	-	-	-	-	-	-	-	-	-	-
	More than one gadget	4.05	4.16	4.52	4.56	4.33	4.62	4.47	3.72	3.23	4.32	3.64	2.85	3.82	3.95	3.70
	$\bar{X}$	3.92	4.07	4.41	4.49	4.29	4.52	4.40	3.63	3.33	4.23	3.57	2.94	3.73	3.71	3.76
	F Test	2.99	1.55	2.53	1.74	0.83	3.18	1.76	1.83	4.08	2.69	1.50	1.70	2.71	9.63	1.16
	Significant	0.03	0.20	0.06	0.16	0.48	0.02	0.15	0.14	0.01	0.05	0.21	0.17	0.04	0.00	0.33
Monthly Internet Budget	Rp 10.000 - Rp 25.000	3.71	4.02	4.31	4.48	4.38	4.60	4.29	3.54	3.54	4.40	3.52	3.33	3.65	3.73	3.92
	Rp 26.000 - Rp 50.000	3.96	4.15	4.45	4.51	4.32	4.50	4.39	3.67	3.29	4.17	3.44	2.99	3.62	3.64	3.65
	Rp 51.000 - Rp 75.000	3.85	4.10	4.37	4.48	4.19	4.51	4.38	3.64	3.29	4.17	3.55	2.84	3.73	3.72	3.73
	>Rp 75.000	4.02	3.99	4.46	4.47	4.37	4.53	4.47	3.62	3.34	4.29	3.71	2.92	3.83	3.74	3.83
	$\bar{X}$	3.92	4.07	4.41	4.49	4.29	4.52	4.40	3.63	3.33	4.23	3.57	2.94	3.73	3.71	3.76
	F Test	1.40	0.86	0.65	0.11	2.05	0.28	1.04	0.18	0.99	1.87	2.25	2.99	1.63	0.35	1.67
	Significant	0.24	0.46	0.58	0.95	0.11	0.84	0.38	0.91	0.40	0.13	0.08	0.03	0.18	0.79	0.17

**Table 7:** Frequency distribution of the respondents’ responses to each item of indicator Variable (*n* = 581).

	Strongly Disagree		Disagree		Neutral		Agree		Strongly Agree		
P	*F*	*%*	*F*	*%*	*F*	*%*	*F*	*%*	*F*	*%*	Average
IS1	9	1.55	7	1.20	81	13.94	191	32.87	293	50.43	4.29
IS2	9	1.55	39	6.71	152	26.16	210	36.14	171	29.43	3.85
IS3	4	0.69	5	0.86	58	9.98	178	30.64	336	57.83	4.44
IS4	7	1.20	17	2.93	75	12.91	201	34.60	281	48.36	4.26
IS5	25	4.30	71	12.22	194	33.39	165	28.40	126	21.69	3.51
IS6	16	2.75	76	13.08	190	32.70	162	27.88	137	23.58	3.56
IS7	15	2.58	46	7.92	161	27.71	219	37.69	140	24.10	3.73
IS8	4	0.69	3	0.52	56	9.64	203	34.94	315	54.22	4.41
IS9	6	1.03	25	4.30	75	12.91	177	30.46	298	51.29	4.27
IA1	11	1.89	18	3.10	77	13.25	211	36.32	264	45.44	4.20
IA2	1	0.17	4	0.69	78	13.43	236	40.62	262	45.09	4.30
IA3	2	0.34	4	0.69	78	13.43	240	41.31	257	44.23	4.28
IA4	1	0.17	6	1.03	61	10.50	237	40.79	276	47.50	4.34
IA5	3	0.52	3	0.52	70	12.05	235	40.45	270	46.47	4.32
CSE1	10	1.72	18	3.10	149	25.65	242	41.65	162	27.88	3.91
CSE2	17	2.93	64	11.02	233	40.10	177	30.46	90	15.49	3.45
CSE3	25	4.30	83	14.29	242	41.65	175	30.12	56	9.64	3.27
CSE4	19	3.27	72	12.39	233	40.10	177	30.46	80	13.77	3.39
CSE5	64	11.02	119	20.48	272	46.82	91	15.66	35	6.02	2.85
R1	25	4.30	31	5.34	131	22.55	175	30.12	219	37.69	3.92
R2	10	1.72	18	3.10	110	18.93	224	38.55	219	37.69	4.07
R3	6	1.03	17	2.93	58	9.98	151	25.99	349	60.07	4.41
R4	2	0.34	4	0.69	51	8.78	177	30.46	347	59.72	4.49
R5	5	0.86	5	0.86	70	12.05	236	40.62	265	45.61	4.29
R6	2	0.34	5	0.86	40	6.88	175	30.12	359	61.79	4.52
E1	2	0.34	4	0.69	55	9.47	216	37.18	304	52.32	4.40
E2	24	4.13	37	6.37	216	37.18	155	26.68	149	25.65	3.63
E3	21	3.61	63	10.84	278	47.85	144	24.78	75	12.91	3.33
E4	1	0.17	8	1.38	88	15.15	246	42.34	238	40.96	4.23
E5	19	3.27	36	6.20	221	38.04	206	35.46	99	17.04	3.57
P1	57	9.81	132	22.72	228	39.24	116	19.97	48	8.26	2.94
P2	15	2.58	18	3.10	197	33.91	231	39.76	120	20.65	3.73
P3	13	2.24	41	7.06	179	30.81	217	37.35	131	22.55	3.71
P4	10	1.72	30	5.16	183	31.50	227	39.07	131	22.55	3.76

**Table 8:** Frequency distribution and correlation between the demographic characteristics of respondents and the Variables of internet skills, internet attitude, computer self efficacy, and digital citizenship (REP).

Characteristic	Sub-Characteristic	Variable	Sub Variable	Strongly Disagree	Disagree	Neutral	Agree	Strongly Agree	N	Max. Score	Score	Mean	%	Category
School Type	Public	Internet Skills		53	174	599	1160	1380	3366	16830	13738	4.08	81.63	Very High
		Internet Attitude		13	18	195	748	896	1870	9350	8106	4.33	86.70	Very High
		Computer Self Efficacy		59	215	708	624	264	1870	9350	6429	3.44	68.76	High
		Digital Citizenship	Respect	24	40	251	755	1174	2244	11220	9747	4.34	86.87	Very High
			Educate	30	86	541	637	576	1870	9350	7253	3.88	77.57	High
			Protect	52	143	501	525	275	1496	7480	5316	3.55	71.07	High
	Private	Internet Skills		42	115	443	546	717	1863	9315	7370	3.96	79.12	High
		Internet Attitude		5	17	169	411	433	1035	5175	4355	4.21	84.15	Very High
		Computer Self Efficacy		76	141	421	238	159	1035	5175	3368	3.25	65.08	High
		Digital Citizenship	Respect	26	40	209	383	584	1242	6210	5185	4.17	83.49	Very High
			Educate	37	62	317	330	289	1035	5175	3877	3.75	74.92	High
			Protect	43	78	286	266	155	828	4140	2896	3.50	69.95	High
Gender	Male	Internet Skills		18	59	256	358	605	1296	6480	5361	4.14	82.73	Very High
		Internet Attitude		6	9	104	252	349	720	3600	3089	4.29	85.81	Very High
		Computer Self Efficacy		29	57	233	240	161	720	3600	2607	3.62	72.42	High
		Digital Citizenship	Respect	9	19	145	275	416	864	4320	3662	4.24	84.77	Very High
			Educate	16	47	209	237	211	720	3600	2740	3.81	76.11	High
			Protect	20	57	206	163	130	576	2880	2054	3.57	71.32	High
	Female	Internet Skills		77	230	786	1348	1492	3933	19665	15747	4.00	80.08	Very High
		Internet Attitude		12	26	260	907	980	2185	10925	9372	4.29	85.78	Very High
		Computer Self Efficacy		106	299	896	622	262	2185	10925	7190	3.29	65.81	High
		Digital Citizenship	Respect	41	61	315	863	1342	2622	13110	11270	4.30	85.96	Very High
			Educate	51	101	649	730	654	2185	10925	8390	3.84	76.80	High
			Protect	75	164	581	628	300	1748	8740	6158	3.52	70.46	High
Age of Respondents	16	Internet Skills		60	159	530	817	999	2565	12825	10231	3.99	79.77	High
		Internet Attitude		13	15	181	577	639	1425	7125	6089	4.27	85.46	Very High
		Computer Self Efficacy		66	195	591	388	185	1425	7125	4706	3.30	66.05	High
		Digital Citizenship	Respect	27	40	233	580	830	1710	8550	7276	4.25	85.10	Very High
			Educate	38	68	420	467	432	1425	7125	5462	3.83	76.66	High
			Protect	49	113	399	373	206	1140	5700	3994	3.50	70.07	High
	17	Internet Skills		34	120	449	768	942	2313	11565	9403	4.07	81.31	Very High
		Internet Attitude		4	17	142	521	601	1285	6425	5553	4.32	86.43	Very High
		Computer Self Efficacy		59	147	464	422	193	1285	6425	4398	3.42	68.45	High
		Digital Citizenship	Respect	17	34	189	486	816	1542	7710	6676	4.33	86.59	Very High
			Educate	26	64	379	444	372	1285	6425	4927	3.83	76.68	High
			Protect	43	92	346	364	183	1028	5140	3636	3.54	70.74	High
	18	Internet Skills		1	10	57	112	135	315	1575	1315	4.17	83.49	Very High
		Internet Attitude		1	3	33	55	83	175	875	741	4.23	84.69	Very High
		Computer Self Efficacy		10	14	67	47	37	175	875	612	3.50	69.94	High
		Digital Citizenship	Respect	6	6	36	64	98	210	1050	872	4.15	83.05	Very High
			Educate	3	16	51	49	56	175	875	664	3.79	75.89	High
			Protect	3	16	39	50	32	140	700	512	3.66	73.14	High
	19	Internet Skills		0	0	5	8	14	27	135	117	4.33	86.67	Very High
		Internet Attitude		0	0	8	4	3	15	75	55	3.67	73.33	High
		Computer Self Efficacy		0	0	6	4	5	15	75	59	3.93	78.67	High
		Digital Citizenship	Respect	0	0	2	8	8	18	90	78	4.33	86.67	Very High
			Educate	0	0	4	7	4	15	75	60	4.00	80.00	Very High
			Protect	0	0	3	3	6	12	60	51	4.25	85.00	Very High
	>20	Internet Skills		0	0	1	1	7	9	45	42	4.67	93.33	Very High
		Internet Attitude		0	0	0	2	3	5	25	23	4.60	92.00	Very High
		Computer Self Efficacy		0	0	1	1	3	5	25	22	4.40	88.00	Very High
		Digital Citizenship	Respect	0	0	0	0	6	6	30	30	5.00	100.00	Very High
			Educate	0	0	4	0	1	5	25	17	3.40	68.00	High
			Protect	0	0	0	1	3	4	20	19	4.75	95.00	Very High
Parents’ Educational Background	Elementary School	Internet Skills		15	41	160	224	226	666	3330	2603	3.91	78.17	High
		Internet Attitude		1	3	53	173	140	370	1850	1558	4.21	84.22	Very High
		Computer Self Efficacy		23	46	152	108	41	370	1850	1208	3.26	65.30	High
		Digital Citizenship	Respect	2	10	57	151	224	444	2220	1917	4.32	86.35	Very High
			Educate	8	17	132	118	95	370	1850	1385	3.74	74.86	High
			Protect	7	21	110	107	51	296	1480	1062	3.59	71.76	High
	Junior High School	Internet Skills		22	62	231	300	285	900	4500	3464	3.85	76.98	High
		Internet Attitude		2	4	76	205	213	500	2500	2123	4.25	84.92	Very High
		Computer Self Efficacy		32	90	210	129	39	500	2500	1553	3.11	62.12	High
		Digital Citizenship	Respect	11	18	89	189	293	600	3000	2535	4.23	84.50	Very High
			Educate	12	21	174	150	143	500	2500	1891	3.78	75.64	High
			Protect	20	38	134	134	74	400	2000	1404	3.51	70.20	High
	Senior High School	Internet Skills		24	131	440	736	874	2205	11025	8920	4.05	80.91	Very High
		Internet Attitude		7	23	163	490	542	1225	6125	5212	4.25	85.09	Very High
		Computer Self Efficacy		46	129	488	394	168	1225	6125	4184	3.42	68.31	High
		Digital Citizenship	Respect	14	30	200	510	716	1470	7350	6294	4.28	85.63	Very High
			Educate	18	52	352	437	366	1225	6125	4756	3.88	77.65	High
			Protect	30	82	344	352	172	980	4900	3494	3.57	71.31	High
	Bachelor's Degree	Internet Skills		30	48	179	393	565	1215	6075	5060	4.16	83.29	Very High
		Internet Attitude		7	5	59	248	356	675	3375	2966	4.39	87.88	Very High
		Computer Self Efficacy		29	72	242	190	142	675	3375	2369	3.51	70.19	High
		Digital Citizenship	Respect	20	16	99	250	425	810	4050	3474	4.29	85.78	Very High
			Educate	23	49	167	229	207	675	3375	2573	3.81	76.24	High
			Protect	30	65	167	173	105	540	2700	1878	3.48	69.56	High
	Master's Degree	Internet Skills		4	7	32	44	129	216	1080	935	4.33	86.57	Very High
		Internet Attitude		1	0	10	42	67	120	600	534	4.45	89.00	Very High
		Computer Self Efficacy		5	18	35	39	23	120	600	417	3.48	69.50	High
		Digital Citizenship	Respect	3	5	14	33	89	144	720	632	4.39	87.78	Very High
			Educate	3	9	33	31	44	120	600	464	3.87	77.33	High
			Protect	7	13	30	24	22	96	480	329	3.43	68.54	High
	Doctoral Degree	Internet Skills		0	0	0	9	18	27	135	126	4.67	93.33	Very High
		Internet Attitude		0	0	3	1	11	15	75	68	4.53	90.67	Very High
		Computer Self Efficacy		0	1	2	2	10	15	75	66	4.40	88.00	Very High
		Digital Citizenship	Respect	0	1	1	5	11	18	90	80	4.44	88.89	Very High
			Educate	3	0	0	2	10	15	75	61	4.07	81.33	Very High
			Protect	1	2	2	1	6	12	60	45	3.75	75.00	High
Frequency of Internet Usage	1-3 hours a day	Internet Skills		1	13	38	62	39	153	765	584	3.82	76.34	High
		Internet Attitude		0	1	19	40	25	85	425	344	4.05	80.94	Very High
		Computer Self Efficacy		2	7	47	14	15	85	425	288	3.39	67.76	High
		Digital Citizenship	Respect	1	3	29	34	35	102	510	405	3.97	79.41	High
			Educate	4	5	31	30	15	85	425	302	3.55	71.06	High
			Protect	1	5	22	27	13	68	340	250	3.68	73.53	High
	4-6 hours a day	Internet Skills		23	72	324	445	486	1350	6750	5349	3.96	79.24	High
		Internet Attitude		5	8	118	320	299	750	3750	3150	4.20	84.00	Very High
		Computer Self Efficacy		27	103	306	218	96	750	3750	2503	3.34	66.75	High
		Digital Citizenship	Respect	12	22	132	288	446	900	4500	3834	4.26	85.20	Very High
			Educate	21	39	263	227	200	750	3750	2796	3.73	74.56	High
			Protect	27	59	209	205	100	600	3000	2092	3.49	69.73	High
	7-9 hours a day	Internet Skills		36	113	357	656	750	1912	9560	7707	4.03	80.62	Very High
		Internet Attitude		8	12	132	468	450	1070	5350	4550	4.25	85.05	Very High
		Computer Self Efficacy		46	123	430	326	145	1070	5350	3611	3.37	67.50	High
		Digital Citizenship	Respect	18	28	156	464	618	1284	6420	5488	4.27	85.48	Very High
			Educate	19	59	315	393	284	1070	5350	4074	3.81	76.15	High
			Protect	29	69	278	327	153	856	4280	3074	3.59	71.82	High
	>9 hours a day	Internet Skills		42	94	375	649	1021	2181	10905	9056	4.15	83.04	Very High
		Internet Attitude		5	14	95	331	555	1000	5000	4417	4.42	88.34	Very High
		Computer Self Efficacy		60	123	346	304	167	1000	5000	3395	3.40	67.90	High
		Digital Citizenship	Respect	19	27	143	352	659	1200	6000	5205	4.34	86.75	Very High
			Educate	23	45	249	317	366	1000	5000	3958	3.96	79.16	High
			Protect	38	88	278	232	164	800	4000	2796	3.50	69.90	High
Gadget Used	Smartphone	Internet Skills		63	219	696	950	1105	3033	15165	11914	3.93	78.56	High
		Internet Attitude		9	25	233	680	738	1685	8425	7168	4.25	85.08	Very High
		Computer Self Efficacy		100	260	677	461	187	1685	8425	5430	3.22	64.45	High
		Digital Citizenship	Respect	30	61	314	654	963	2022	10110	8525	4.22	84.32	Very High
			Educate	43	90	531	533	488	1685	8425	6388	3.79	75.82	High
			Protect	57	129	483	436	243	1348	6740	4723	3.50	70.07	High
	Tablet	Internet Skills		0	0	0	1	8	9	45	44	4.89	97.78	Very High
		Internet Attitude		0	0	0	0	5	5	25	25	5.00	100.00	Very High
		Computer Self Efficacy		0	0	0	2	3	5	25	23	4.60	92.00	Very High
		Digital Citizenship	Respect	0	0	0	0	6	6	30	30	5.00	100.00	Very High
			Educate	0	0	0	0	5	5	25	25	5.00	100.00	Very High
			Protect	0	1	0	0	3	4	20	17	4.25	85.00	Very High
	Laptop	Internet Skills		1	1	6	11	17	36	180	150	4.17	83.33	Very High
		Internet Attitude		0	0	3	8	9	20	100	86	4.30	86.00	Very High
		Computer Self Efficacy		0	0	3	12	5	20	100	82	4.10	82.00	Very High
		Digital Citizenship	Respect	0	1	1	10	12	24	120	105	4.38	87.50	Very High
			Educate	0	0	5	5	10	20	100	85	4.25	85.00	Very High
			Protect	2	0	8	5	1	16	80	51	3.19	63.75	High
	More than one gadget	Internet Skills		38	74	405	856	1120	2493	12465	10425	4.18	83.63	Very High
		Internet Attitude		9	10	128	471	577	1195	5975	5182	4.34	86.73	Very High
		Computer Self Efficacy		35	96	449	387	228	1195	5975	4262	3.57	71.33	High
		Digital Citizenship	Respect	20	18	145	474	777	1434	7170	6272	4.37	87.48	Very High
			Educate	24	58	322	429	362	1195	5975	4632	3.88	77.52	High
			Protect	36	91	296	350	183	956	4780	3421	3.58	71.57	High
Monthly Internet Budget	Rp 10,000 - Rp 25,000	Internet Skills		12	24	81	117	198	432	2160	1761	4.08	81.53	Very High
		Internet Attitude		3	1	27	91	118	240	1200	1040	4.33	86.67	Very High
		Computer Self Efficacy		18	26	74	70	52	240	1200	832	3.47	69.33	High
		Digital Citizenship	Respect	9	11	42	63	163	288	1440	1224	4.25	85.00	Very High
			Educate	12	7	68	69	84	240	1200	926	3.86	77.17	High
			Protect	5	19	58	65	45	192	960	702	3.66	73.13	High
	Rp 26,000 - Rp 50,000	Internet Skills		18	72	293	443	470	1296	6480	5163	3.98	79.68	High
		Internet Attitude		1	8	79	325	307	720	3600	3089	4.29	85.81	Very High
		Computer Self Efficacy		31	95	307	205	82	720	3600	2372	3.29	65.89	High
		Digital Citizenship	Respect	7	15	110	299	433	864	4320	3728	4.31	86.30	Very High
			Educate	13	32	236	250	189	720	3600	2730	3.79	75.83	High
			Protect	27	46	217	199	87	576	2880	2001	3.47	69.48	High
	Rp 51,000 - Rp 75,000	Internet Skills		28	111	376	695	716	1926	9630	7738	4.02	80.35	Very High
		Internet Attitude		6	14	143	452	455	1070	5350	4546	4.25	84.97	Very High
		Computer Self Efficacy		44	121	418	355	132	1070	5350	3620	3.38	67.66	High
		Digital Citizenship	Respect	17	29	179	448	611	1284	6420	5459	4.25	85.03	Very High
			Educate	20	58	325	373	294	1070	5350	4073	3.81	76.13	High
			Protect	31	97	278	308	142	856	4280	3001	3.51	70.12	High
	>Rp 75,000	Internet Skills		37	82	292	451	713	1575	7875	6446	4.09	81.85	Very High
		Internet Attitude		8	12	115	291	449	875	4375	3786	4.33	86.54	Very High
		Computer Self Efficacy		42	114	330	232	157	875	4375	2973	3.40	67.95	High
		Digital Citizenship	Respect	17	25	129	328	551	1050	5250	4521	4.31	86.11	Very High
			Educate	22	51	229	275	298	875	4375	3401	3.89	77.74	High
			Protect	32	59	234	219	156	700	3500	2508	3.58	71.66	High

Table 1 presents the characteristics of the respondents based on the demography and socio-economy of the respondents. Previous studies denote users’ individual background such as gender, age, frequency of internet usage, and parents’ educational level have influential relationship with digital citizenship. Choi et al. [1] reported that individual background has narrowly impacted to digital literacy. Hargittai [2] found that parental education has no longer related to digital tools use but it is imperative in explaining variation in user skill. Besides, Wang and Xing [3] examined that parents' socioeconomic status includes parents' income and education level as a significant predictor of digital citizenship.

Meanwhile, Table 2 describes the frequency distribution for each question item in the questionnaire. In Table 3, the data on the relationship between the demographic characteristics of the respondents and the variables are presented in a contingency table to show the interactions that occur between the demographic and socio-economic variables of the respondents and the variables of internet skills, internet attitude, computer self-efficacy, and digital citizenship which were reviewed from 3 components including respect, educate and protect in details.

## 2 Experimental Design, Materials and Methods

The context in this study is digital citizenship competence of senior high school students. The research samples came from 12 schools located in Central Java, Indonesia which were selected by probability sampling, in which the samples were allowed to choose based on random convenient sample to be used in research [4]. Because the pandemic of Covid-19 forced schools to close, the data collection was carried out through an online questionnaire. The questionnaires were distributed to respondents according to the criteria determined by the researcher using the Google Form platform. This was carried out with the aim of being able to measure the digital citizenship readiness of students in accordance with the Nine Elements parameter of digital citizenship including protection (protect), education (educate), and respect that was developed by Al-Zahrani [5] based on the assumption of Ribble [6]. Jones and Mitchell [8] also developed Digital Citizenship Scale (DCS) based on the criteria of online respect and online civic engagement with the total of 11 question items using 5-point scale from “not all like me” to “very much like me”. In this study, the point scale was the same as the DCS by Al-Zahrani [5] with 5-point Likert Scale (5 = Strongly Agree, 1 = Strongly Disagree). In addition, the question items for the variables of internet attitude and computer self-efficacy were also based on the study by Al-Zahrani [5]. As for the variable of internet skills, it was adapted from van Deursen et al. [7].

A total of 581 responses were received by the researcher, and all of them met the criteria for the next stage in the form of statistical analysis. In the aspect of demographic characteristics of the students, the descriptive statistical test was carried out with analytical technique using one-way ANOVA to determine the relationship and the influence of these demographic factors on the variables of internet skills, internet attitudes, computer self-efficacy, and the basic components that form the digital citizenship. Meanwhile, the analysis of the reliability and validity of the instrument was carried out using the Structural Equation Model (SEM) analysis technique with the SmartPLS version 3 program.

On the demographic characteristics of the respondents, measurements were made regarding the aspects used as parameters for the level of digital citizenship of the students. Individual demographic data is a potential source of data related to the students' knowledge, attitude, skills, and practices in utilizing digital technology within their daily lives which were analyzed using frequency and percentage. One-Way ANOVA was carried out to determine the relationship between different experiences and expertise in using computers and how they utilize it to facilitate activities in their daily lives. In Table 8, the category level of the respondents is described in each of the tested variables. The majority of the distribution of responses fell under the high category in which the variable of internet attitude obtained 54.22%, that of internet skills obtained 56.28% which was included in the high category, and the percentage of frequency distribution of the respondents' responses was 54.56% for the variable of protect, 53.18% for computer self-efficacy and 51.64% for educate, thus the three of them belonged to the medium category. Meanwhile, the variable of respect fell under the very high category with 57.31%. Therefore, the respondents had a very high level of respect in utilizing computers and accessing internet within their daily lives.

## Ethics Statement

Universitas Muhammadiyah Surakarta has responsibility for this project. The study process has been approved and adheres to the ethical guidelines and regulations the organisation in charge. The study used a tix box in the online consent form for underage participants to discuss the study with their parents and understand the study procedure, risks, and benefits to allow their children to be included. The study was conducted with the agreement and the volunteer of school managers and local administrators. All respondents were informed relate to the research before conducting the survey. The online survey was written anonymously to guarantee the confidentiality of their personal data.

## CRediT authorship contribution statement

**Wibowo Heru Prasetiyo:** Conceptualization, Methodology, Investigation, Writing – review & editing. **Noor Banu Mahadir Naidu:** Conceptualization, Resources, Writing – review & editing. **Beti Indah Sari:** Formal analysis, Validation, Writing – original draft. **Rochman Hadi Mustofa:** Formal analysis, Resources, Validation. **Naillysa Rahmawati:** Investigation, Data curation. **Gilang Pambudi Adi Wijaya:** Investigation, Resources. **Obby Taufik Hidayat:** Investigation, Resources.

## Declaration of Competing Interest

The authors declare that they have not known competing financial interests or personal relationships, which have, or could be perceived to have, influenced the work reported in this article.

## Appendix Supplementary materials

Image, application 1

## References

[1] Choi M., Cristol D., Gimbert B. (2018). Teachers as digital citizens: The influence of individual backgrounds, internet use and psychological characteristics on teachers’ levels of digital citizenship. Comput. Educ..

[2] Hargittai E. (2010). Digital Na(t)ives? Variation in internet skills and uses among members of the “net Generation. Sociol. Inq..

[3] Wang X., Xing W. (2018). Exploring the influence of parental involvement and socioeconomic status on teen digital citizenship: a path modeling approach. Educ. Technol. Soc..

[4] Mertens D.M. (2015).

[5] Al-Zahrani A. (2015). Toward digital citizenship: examining factors affecting participation and involvement in the internet society among higher education students. Int Educ Stud.

[6] Ribble M. (2015).

[7] van Deursen A.J.A.M., Helsper E.J., Eynon R. (2016). Development and validation of the internet skills scale (ISS). Inf. Commun. Soc..

[8] Jones L.M., Mitchell K.J. (2016). Defining and measuring youth digital citizenship. New Media Soc..

