# Supplementary material for: Survey data of internet skills, internet attitudes, computer self-efficacy, and digital citizenship among students in Indonesia
Source: Data Brief. 2021 Nov 16;39:107569. doi: 10.1016/j.dib.2021.107569 (PMC8605400; doi:10.1016/j.dib.2021.107569)
Supplement: Supplementary file 1 [file mmc1.docx]

**RESEARCH QUESTIONNAIRES**

1. Directions: Put a tick (**✓**) in the column that you choose.

Demographic Variables

1. Male/Female:

- Male
- Female

1. Age:

- 16 years old
- 17 years old
- 18 years old
- 19 years old
- 20 years old
- 21 years old

1. Parents’ Education:

- Elementary school
- Junior high school
- Senior high school
- Bachelor
- Master
- Doctor

1. Length of Internet Use (in a day):

- 1-3 hours
- 4-6 hours
- 7-9 hours
- > 9 hours

1. Personal Devices:

- Smartphone
- Tablet
- Laptop
- Personal Computer

1. Internet Budget (in a month):

- Rp. 10.000 – Rp. 25.000
- Rp. 26.000 – Rp. 50.000
- Rp. 51.000 – Rp. 75.000
- > Rp. 75.000

1. Directions: Please respond to the statements below using the following scale by circling the number that best represents your response. Circle only one answer; do not circle between numbers or indicate a fraction or a range.

| **Variabel** | **Items** | **1-Strongly Disagree** | **2-Disagree** | **3-Neutral** | **4-Agree** | **5-Strongly Agree** |
| --- | --- | --- | --- | --- | --- | --- |
| Internet Skills  (van Deursen et al., 2016) | 1. I feel I can download videos/photos from the internet | 1 | 2 | 3 | 4 | 5 |
|  | 1. I feel I can use basic computer shortcut keys | 1 | 2 | 3 | 4 | 5 |
|  | 1. I feel I can manage passwords in android/iPhone | 1 | 2 | 3 | 4 | 5 |
|  | 1. I feel I can connect my device with Wi-Fi | 1 | 2 | 3 | 4 | 5 |
|  | 1. I am having difficulty finding specific keywords using search engine | 1 | 2 | 3 | 4 | 5 |
|  | 1. I am having difficulty checking previous website visits | 1 | 2 | 3 | 4 | 5 |
|  | 1. I feel I am capable of editing photos/videos | 1 | 2 | 3 | 4 | 5 |
|  | 1. I feel I can reinstall a program on a smartphone/laptop | 1 | 2 | 3 | 4 | 5 |
|  | 1. I feel I am capable of checking data usage on a smartphone | 1 | 2 | 3 | 4 | 5 |
| Internet Attitude  (Al-Zahrani, 2015) | 1. Internet is helpful to eliminate a lot of tedious work | 1 | 2 | 3 | 4 | 5 |
|  | 1. Internet brings us into an advance digital era | 1 | 2 | 3 | 4 | 5 |
|  | 1. Life will be easier and faster with the internet | 1 | 2 | 3 | 4 | 5 |
|  | 1. Internet provides easy, rich, and enjoyable information | 1 | 2 | 3 | 4 | 5 |
|  | 1. Internet enhances our standard of living | 1 | 2 | 3 | 4 | 5 |
| Computer Self-Efficacy  (Al-Zahrani, 2015) | 1. I feel confident working a personal computer | 1 | 2 | 3 | 4 | 5 |
|  | 1. I feel confident installing or reinstalling any programs on my personal computer | 1 | 2 | 3 | 4 | 5 |
|  | 1. I feel confident understanding several terms relating to hardware and software | 1 | 2 | 3 | 4 | 5 |
|  | 1. I feel confident developing my skills within a specific software | 1 | 2 | 3 | 4 | 5 |
|  | 1. I feel confident troubleshooting computer problems | 1 | 2 | 3 | 4 | 5 |
| Digital Citizenship  (Al-Zahrani, 2015)   - Subscale Respect (6 items) | 1. I believe that creating worms or viruses, trojan, and sending spam emails are digital crimes | 1 | 2 | 3 | 4 | 5 |
|  | 1. I believe that technology use might impact digital health | 1 | 2 | 3 | 4 | 5 |
|  | 1. I believe that hacking others’ social media/email, download illegally, and copyright infringement is unethical behaviors | 1 | 2 | 3 | 4 | 5 |
|  | 1. In a digital environment, I believe that everyone should take responsibility for his/her activities | 1 | 2 | 3 | 4 | 5 |
|  | 1. In a digital environment, I believe that all users have equal rights of speak and expression | 1 | 2 | 3 | 4 | 5 |
|  | 1. Smartphone and computer help me to communicate with friends | 1 | 2 | 3 | 4 | 5 |
| - Subscale Educate (5 items) | 1. I take the time to search for information before buying some goods from e-commerce | 1 | 2 | 3 | 4 | 5 |
|  | 1. I prefer to buy on e-commerce than going to the market | 1 | 2 | 3 | 4 | 5 |
|  | 1. I use social media to express my opinion, feeling, and experience | 1 | 2 | 3 | 4 | 5 |
|  | 1. I have been taught the new insight from the internet | 1 | 2 | 3 | 4 | 5 |
|  | 1. I spend my valuable on media social, such as Facebook and Instagram | 1 | 2 | 3 | 4 | 5 |
| - Subscale Protect (5 items) | 1. I regularly change my password on smartphone/computer to protect my privacy | 1 | 2 | 3 | 4 | 5 |
|  | 1. I always visit trusted and harm-free website | 1 | 2 | 3 | 4 | 5 |
|  | 1. I have installed antivirus and internet protection on my computer | 1 | 2 | 3 | 4 | 5 |
|  | 1. I always read the privacy statement before installing new software | 1 | 2 | 3 | 4 | 5 |

**References**

Al-Zahrani, A. (2015). Toward Digital Citizenship: Examining Factors Affecting Participation and Involvement in the Internet Society among Higher Education Students. *International Education Studies*, *8*(12), 203. https://doi.org/10.5539/ies.v8n12p203

van Deursen, A. J. A. M., Helsper, E. J., & Eynon, R. (2016). Development and validation of the Internet Skills Scale (ISS). *Information Communication and Society*, *19*(6), 804–823. https://doi.org/10.1080/1369118X.2015.1078834
